# Supplementary material for: Targeted cross-linker delivery for the in situ mapping of protein conformations and interactions in mitochondria
Source: Nat Commun. 2023 Jun 30;14:3882. doi: 10.1038/s41467-023-39485-3 (PMC10313818; doi:10.1038/s41467-023-39485-3)
Supplement: Supplementary file 1 — Supplementary Information [file 41467_2023_39485_MOESM1_ESM.pdf]

Supplementary Information

Targeted cross-linker delivery for the in situ mapping of protein conformations and interactions in mitochondria

Yuwan Chen[1,2], Wen Zhou[1,2], Yufei Xia[3], Weijie Zhang[1,2], Qun Zhao[1], Xinwei Li[1,4], Hang Gao[1,2], Zhen Liang[1], Guanghui Ma[3], Kaiguang Yang[1]*, Lihua Zhang[1]* & Yukui Zhang[1]

[1] CAS Key Laboratory of Separation Science for Analytical Chemistry, Dalian Institute of Chemical Physics, Chinese Academy of Sciences, Dalian 116023, China.
[2] University of Chinese Academy of Sciences, Beijing 100049, China.
[3] State Key Laboratory of Biochemical Engineering, Institute of Process Engineering, Chinese Academy of Sciences, Beijing 100190, China.
[4] Zhang Dayu School of Chemistry, Dalian University of Technology, Dalian 116024, China.

* ✉ email: yangkaiguang@dicp.ac.cn; lihuazhang@dicp.ac.cn.

Contents

Supplementary Figure 1. The cross-linking effect of the cross-linker in the system was analyzed using SDS-PAGE..... 3
Supplementary Figure 2. The effect of dimethyldioctadecylammonium bromide (DDAB) content in the nanoparticles (NPs) on the percentage of proteins with 2-fold significant changes at the proteome level in the treated HepG2 cells.. 3
Supplementary Figure 3. Synthetic route of DSS functionalized with FITC (DSS-FITC, $C_{39}H_{35}N_3O_{14}$)......... 4
Supplementary Figure 4. ^{1}H-NMR of DSS functionalized with FITC (DSS-FITC, $C_{39}H_{35}N_3O_{14}$) in DMSO-d6 (400 MHz). 4
Supplementary Figure 5. LTQ MS spectrum of DSS functionalized with FITC (DSS-FITC, $C_{39}H_{35}N_3O_{14}$)......... 5
Supplementary Figure 6. Flow cytometry analysis of the labeled nanoparticles (NPs) and unlabeled NPs. 5
Supplementary Figure 7. Control experiments for proving the mitochondria targeted by surface positive charge doping......... 6
Supplementary Figure 8. Characterization of the intracellular spatial distribution of the nanoparticles (NPs). 7
Supplementary Figure 9. Co-localizations of the dimethyldioctadecylammonium bromide (DDAB)@poly (lactic-co-glycolic acid) (PLGA)/Kolliphor EL nanoparticles (NPs)/lysosomes were measured using CLSM at different times. 8
Supplementary Figure 10. Flowchart of nanoparticle (NP)-corona complex preparation......... 9
Supplementary Figure 11. Assessment of nanoparticle (NP)-corona complexes......... 10
Supplementary Figure 12. Evaluation of the effect of CD-MS labeling mitochondrial proteins. 10
Supplementary Figure 13. Analysis of the CD-MS-labeled mitochondrial cross-linked peptides.. 11
Supplementary Figure 14. Pathway analysis of the identified tricarboxylic acid cycle (TCA cycle) proteins using the KEGG mapper. 12
Supplementary Figure 15. Structural match of the detected cross-linked peptides of tricarboxylic acid cycle (TCA cycle) proteins......... 13
Supplementary Figure 16. AlphaFold prediction model. 14
Supplementary Figure 17. Pathway analysis of the identified oxidative phosphorylation (OXPHOS) proteins using KEGG mapper.. 14
Supplementary Figure 18. Analysis of the cross-linked peptides of the oxidative phosphorylation (OXPHOS) proteins identified in all fractionations. 15
Supplementary Figure 19. Analysis of the cross-linked peptides of ATP synthase (complex V) identified in all fractionations......... 15
Supplementary Figure 20. Analysis of the cross-linked peptides of the tricarboxylic acid cycle (TCA cycle) and oxidative phosphorylation (OXPHOS) identified in all fractionations......... 16
Supplementary Figure 21. Structural match of the detected cross-linked peptides of the SLC25 family......... 17
Supplementary Figure 22. Hydrophobic/hydrophilic properties of cross-linkers......... 18

Supplementary Table 1. Size, PDI, and zeta potential variations of the three nanoparticles (NPs) in Fig. 2b. 18
Supplementary Table 2 . In vitro disuccinimidylsuberate (DSS) release experiment of 70DSS-dimethyldioctadecylammonium bromide (DDAB) @ poly (lactic-co-glycolic acid) (PLGA) / nanoparticles (NPs).. 18
Supplementary Table 3. The adsorbed proteins on the CDNP^{D}, CDNP^{DL}, and CDNP^{DLC} surfaces, corresponding to the numbers in Supplementary Fig. 11b.. 19
Supplementary Table 4. A comparison of different methods for studying mitochondrial information......... 19

Supplementary Figures and Tables

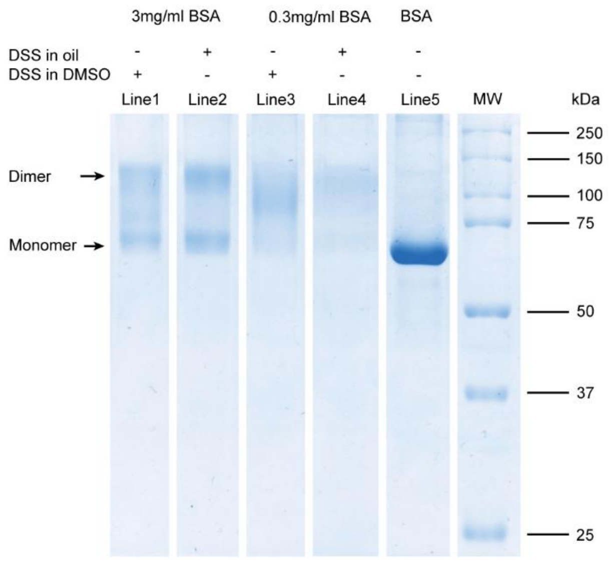

Supplementary Figure 1. The cross-linking effect of the cross-linker in the system was analyzed using SDS-PAGE. The Bovine Serum albumin (BSA) was cross-linked by disuccinimidylsuberate (DSS), which was dissolved in 10% dimethyl sulfoxide (DMSO) (PBS (v/v) (0.01 M, pH = 7.2) or 10% Kolliphor EL oil (PBS (v/v) (0.01 M, pH = 7.2). To represent the intracellular protein concentration, the BSA concentrations were chosen as 3 mg/mL and 0.3 mg/mL. To compare the cross-linking efficiency of the two methods, cross-linked BSA was separated using 12% SDS-PAGE gel (blue), using native BSA as the negative control. The gel was stained with Coomassie brilliant blue. Molecular weight (MW). Source data are provided as a Source Data file.

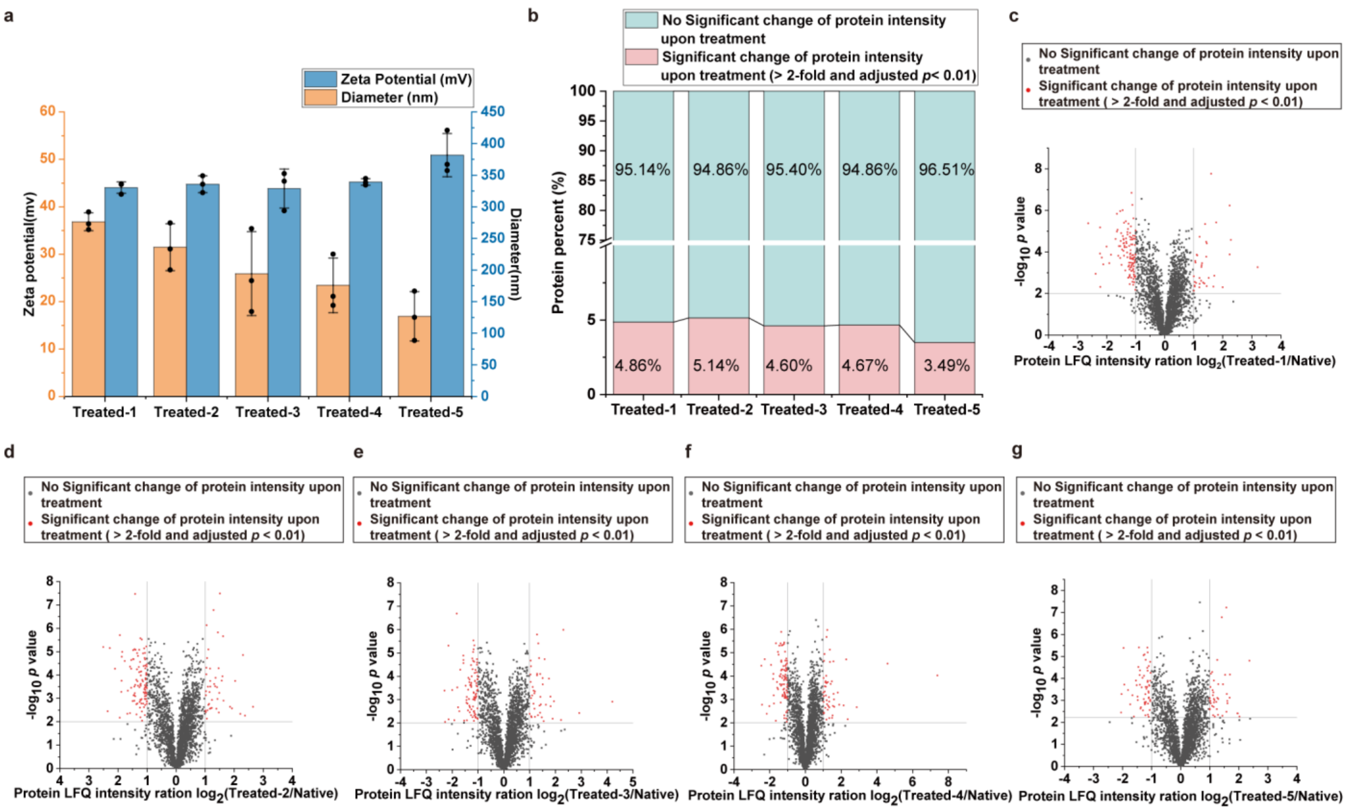

Supplementary Figure 2. The effect of dimethyldioctadecylammonium bromide (DDAB) content in the nanoparticles (NPs) on the percentage of proteins with 2-fold significant changes at the proteome level in the treated HepG2 cells. a Size (blue) and zeta potential (orange) of the disuccinimidylsuberate (DSS)-DDAB@ poly (lactic-co-glycolic acid) (PLGA)/Kolliphor EL NPs with different amounts of DDAB in pure water, as measured using DLS. Data in (a) was represented as mean values ± SD, n = 3 independent experiments. **b** Significant changes in protein abundance. The protein changes at the cellular protein levels in

cells treated with the highest loading efficiency NPs for 6 h were measured using the label-free protein quantification method (Treated-1 to -5). *P* value was calculated by using Student's T-test (adjusted $p < 0.01$). The *P* value was adjusted for multiple tests using FDR (Permutation-based). Protein percent with a significant change in LFQ intensity of more than 2-fold in the treated group compared with the untreated condition were shown as light pink, while the remaining proteins were shown as sky blue. **c–g** Label-free quantification was performed to calculate the changes of protein abundance between the native and different 70DSS-DDAB@PLGA/Kolliphor EL NP-treated HepG2 cells. The DDAB content in the different 70DSS-DDAB@PLGA/Kolliphor EL NPs was changed to 80% (Treated-2), 60% (Treated-3), 40% (Treated-4), and 20% (Treated-5) of the initial content (Treated-1), respectively. Proteins with a significant change in LFQ intensity of more than 2-fold in the treated group compared with the untreated condition were shown as red dots, while the remaining proteins were shown as black dots. *P* values was calculated by using Student's T-test (adjusted $p < 0.01$). The *P* values were adjusted for multiple tests using an FDR (Permutation-based). Source data are provided with this paper.

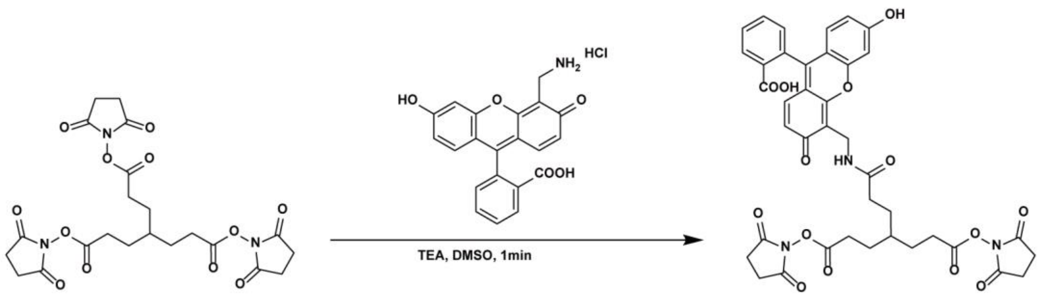

Supplementary Figure 3. Synthetic route of DSS functionalized with FITC (DSS-FITC, $C_{39}H_{35}N_3O_{14}$).

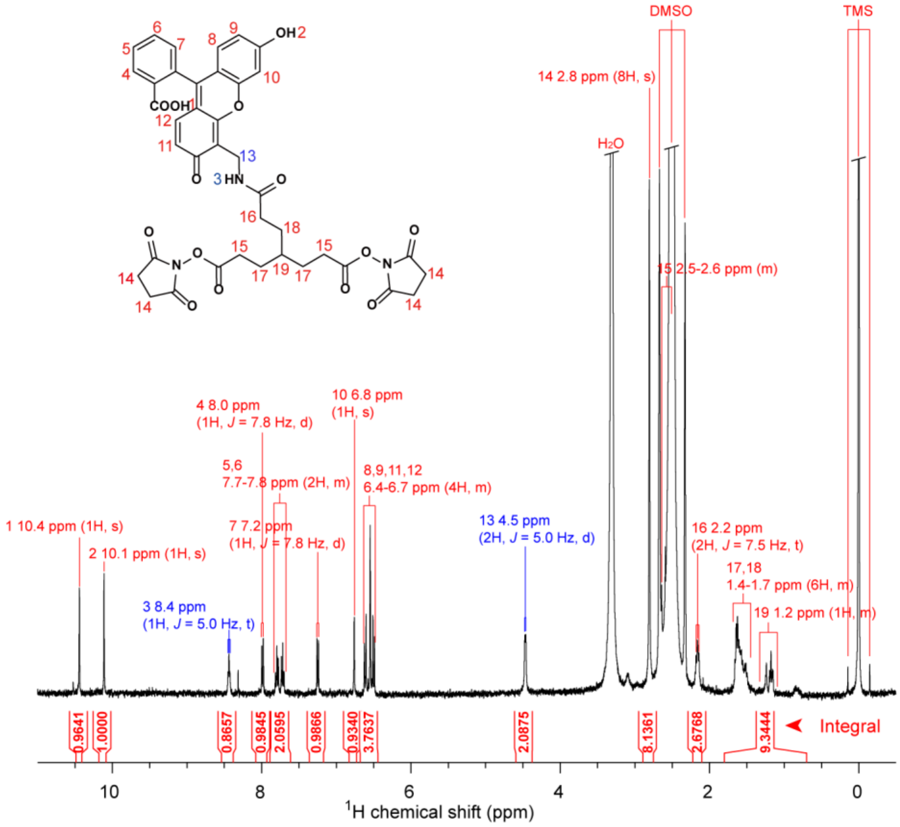

Supplementary Figure 4. ^{1}H-NMR of DSS functionalized with FITC (DSS-FITC, $C_{39}H_{35}N_3O_{14}$) in DMSO-d6 (400 MHz). The hydrogen bonds forming the product were shown in blue, the remaining hydrogen bonds were in red. Source data are provided with this paper.

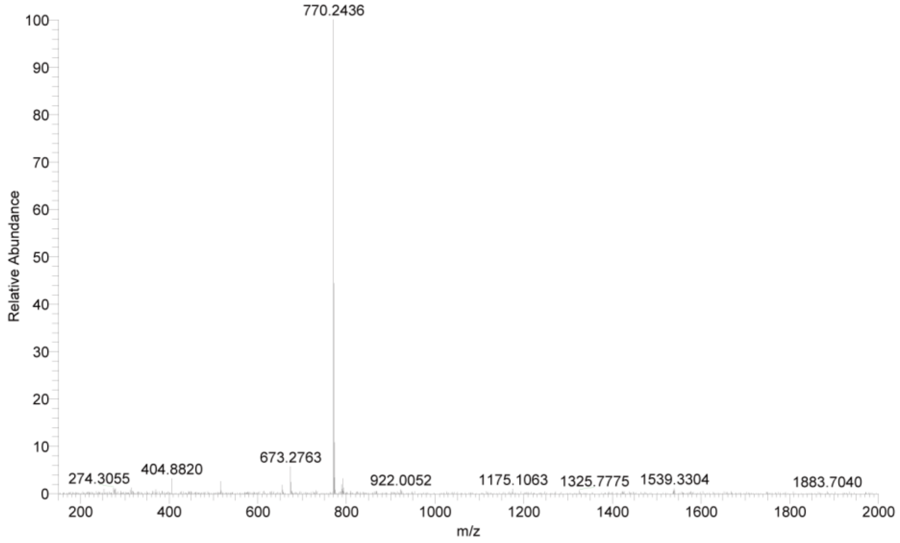

Supplementary Figure 5. LTQ MS spectrum of DSS functionalized with FITC (DSS-FITC, $C_{39}H_{35}N_3O_{14}$). Source data are provided with this paper.

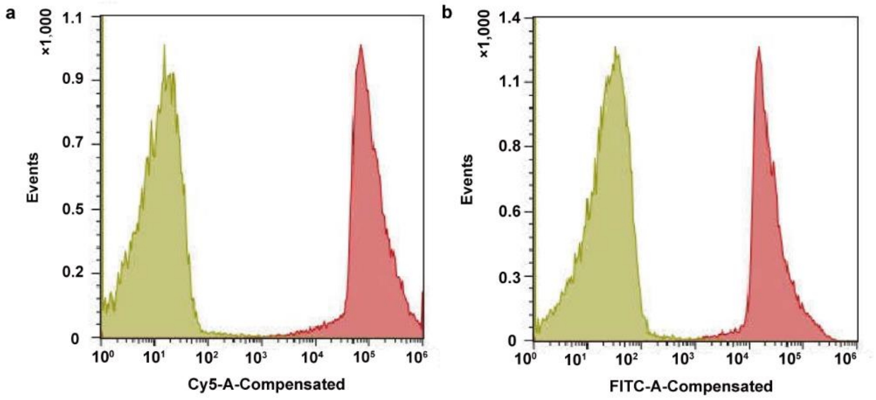

Supplementary Figure 6. Flow cytometry analysis of the labeled nanoparticles (NPs) and unlabeled NPs. **a** Encapsulation of Cy5 into the DDAB@PLGA/Kolliphor EL NPs (red channel). For the negative control, the DDAB@PLGA/Kolliphor EL NPs (grey-green channel) were not labeled with cy5. Flow cytometry analysis was performed using an SH800S cell sorter (Sony Biotechnology). **b** Encapsulation of DSS-FITC into the DDAB@PLGA/Kolliphor EL NPs (red channel). For the negative control, the DDAB@PLGA/Kolliphor EL NPs (grey-green channel) were not labeled with DSS-FITC. Flow cytometry analysis was performed using an SH800S cell sorter (Sony Biotechnology). Source data are provided with this paper.

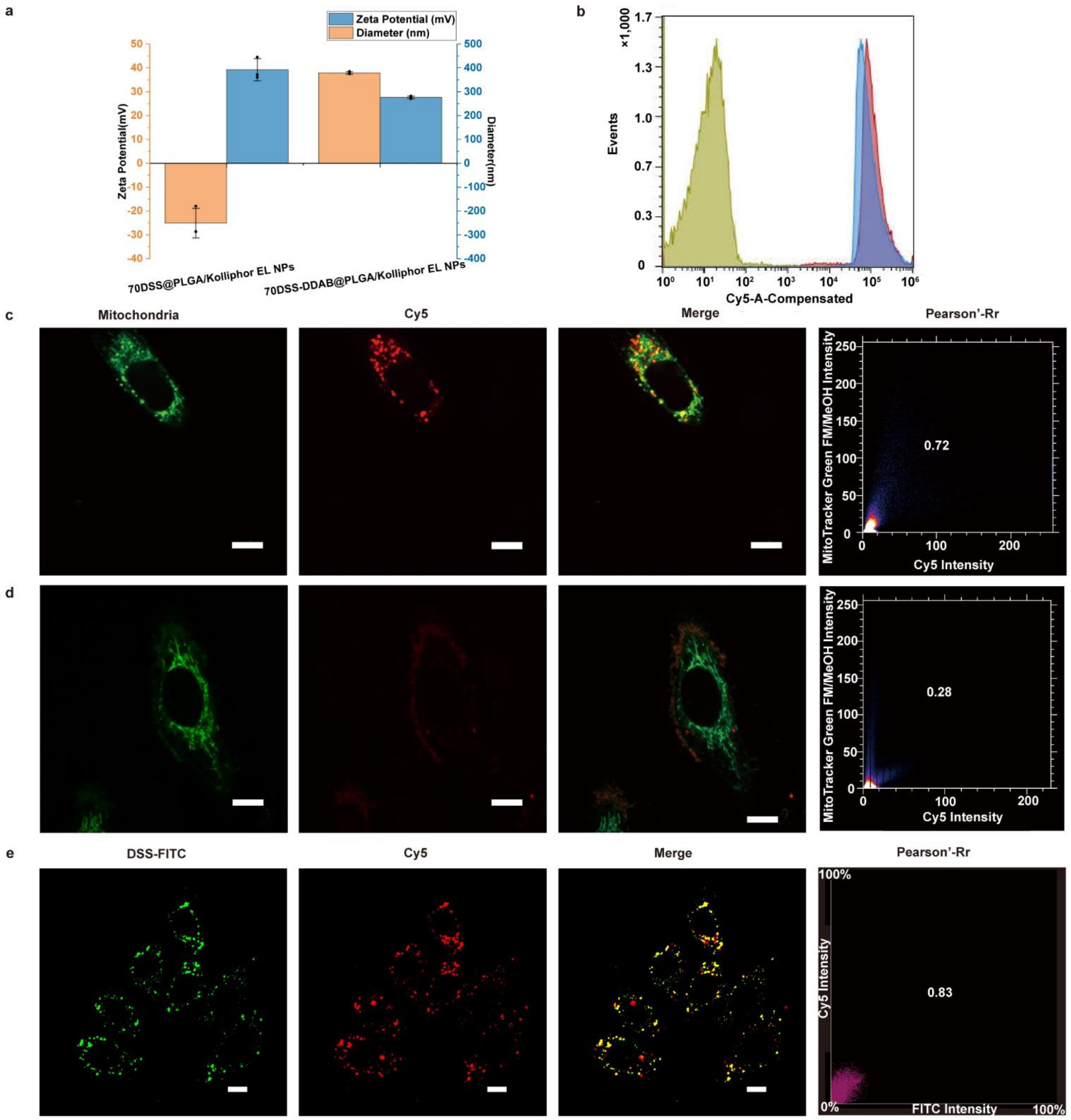

Supplementary Figure 7. Control experiments for proving the mitochondria targeted by surface positive charge doping. a Size (orange) and zeta potential (blue) of the 70 disuccinimidylsuberate (DSS)-dimethyldioctadecylammonium bromide (DDAB) @ poly (lactic-co-glycolic acid) (PLGA)/Kolliphor EL nanoparticles (NPs) and 70DSS@PLGA/Kolliphor EL NPs in water measured using DLS. Data in (a) was represented as mean values ± SD, n = 3 independent experiments. **b** Flow cytometry determination of the amounts of the DDAB@PLGA/Kolliphor EL NPs (grey green), cy5-labeled DDAB@PLGA/Kolliphor EL NPs (blue) and cy5-labeled PLGA/Kolliphor EL NPs (red). **c** Co-localizations of the DDAB@PLGA/Kolliphor EL NPs/mitochondria, as measured using Revolution WD (Scale bars = 10μm). The DDAB@PLGA/Kolliphor EL NPs and mitochondria were labeled with Cy5 (red) and MitoTracker Green (green), respectively. **d** Co-localizations of the PLGA/Kolliphor EL NPs and mitochondria, as measured using Revolution WD (Scale bars = 10μm). The PLGA/Kolliphor EL NPs and mitochondria were labeled with Cy5 (red) and MitoTracker Green (green), respectively. **e** Co-localizations of DSS-FITC (green), which was embedded in the cy5-labeled DDAB@PLGA/Kolliphor EL NPs and cy5-labeled DDAB@PLGA/Kolliphor EL NPs (red), as measured using CLSM. Scale bars = 10μm. Confocal images e was processed by the deconvolution method in NIS-Elements AR 5.20.00 (Nikon). Confocal image c-e from co-localization experiments is representative of biological duplicates. Source data are provided with this paper.

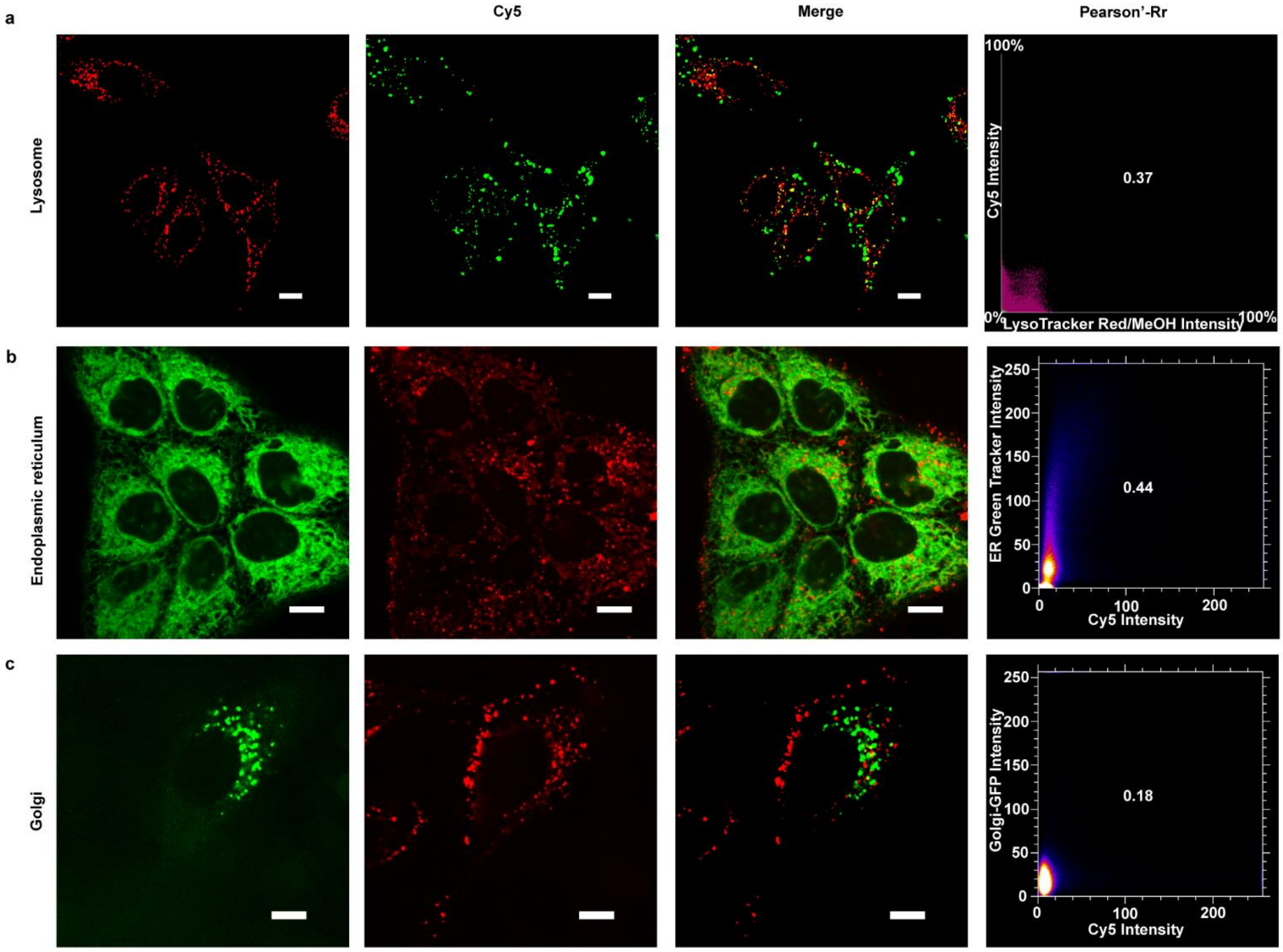

Supplementary Figure 8. Characterization of the intracellular spatial distribution of the nanoparticles (NPs). a Co-localizations of the cy5-labeled dimethyldioctadecylammonium bromide (DDAB)@poly (lactic-co-glycolic acid) (PLGA)/Kolliphor EL NPs and lysosomes, as measured using CLSM. Lysosomes were labeled with LysoTracker Red (red). Scale bars = 10 µm. **b** Co-localizations of the cy5-labeled DDAB@PLGA/Kolliphor EL NPs (red) and endoplasmic reticulum, as measured using Revolution XD. The endoplasmic reticulum was labeled with ER-Tracker Green (green). Scale bars = 10 µm. **c** Co-localizations of the cy5-labeled DDAB@PLGA/Kolliphor EL NPs (red) and Golgi apparatus, as measured using Revolution XD. The Golgi apparatus was labeled with Golgi-GFP (green). Scale bars = 10 µm. Confocal images a were processed by the deconvolution method in NIS-Elements AR 5.20.00 (Nikon). Confocal images a-c from co-localization experiments are representatives of biological duplicates. Source data are provided with this paper.

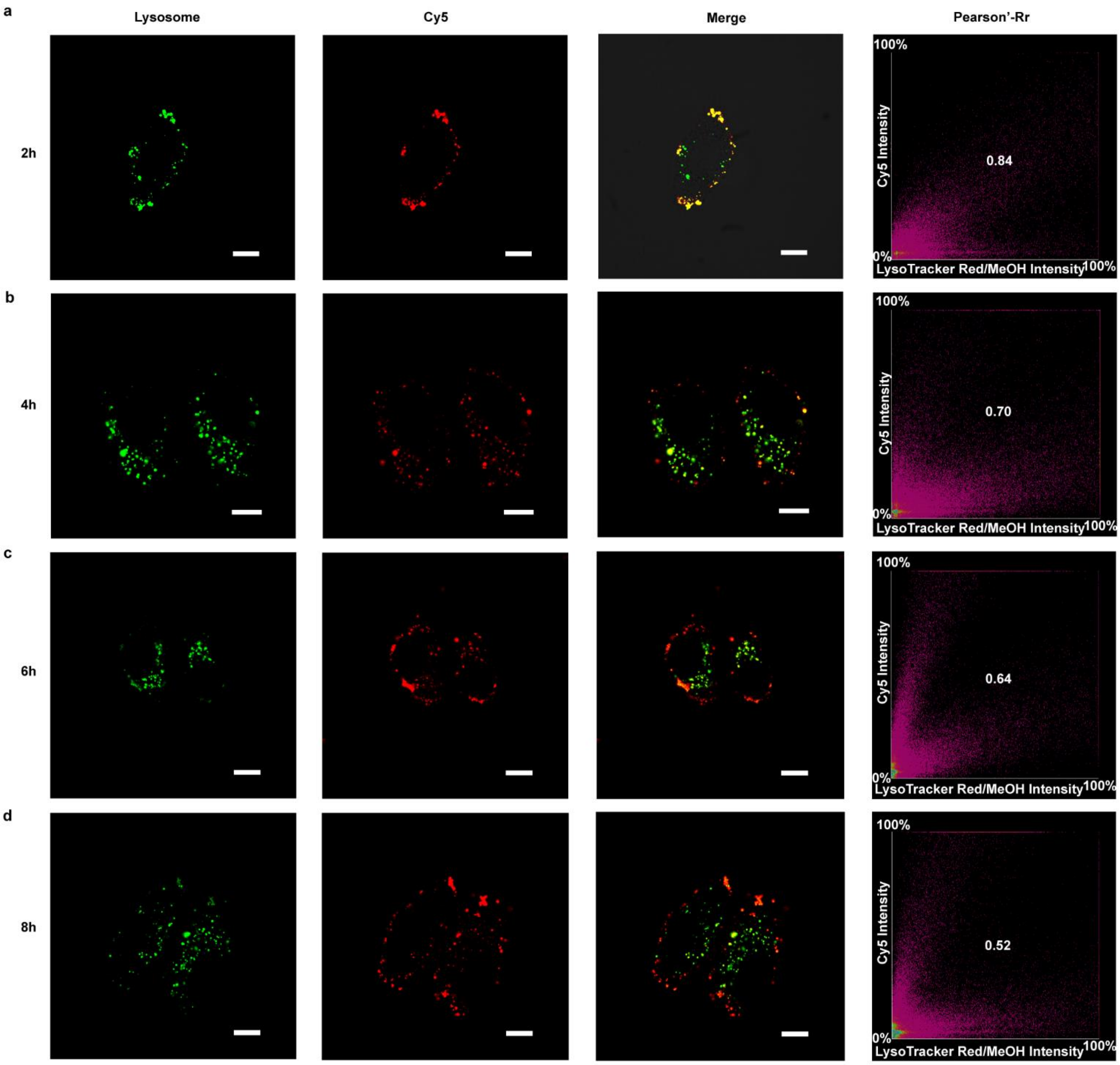

Supplementary Figure 9. Co-localizations of the dimethyldioctadecylammonium bromide (DDAB)@poly (lactic-co-glycolic acid) (PLGA)/Kolliphor EL nanoparticles (NPs)/lysosomes were measured using CLSM at different times. The DDAB@PLGA/Kolliphor EL NPs and lysosomes were labeled with Cy5(red) and LysoTracker Red (green), respectively. **a** The HepG2 cells were co-incubated with DDAB@PLGA/Kolliphor EL NPs for 2 h, **b** 4 h, **c** 6 h, and **d** 8 h, as observed with CLSM. Scale bars = 10μm. Source data are provided with this paper.

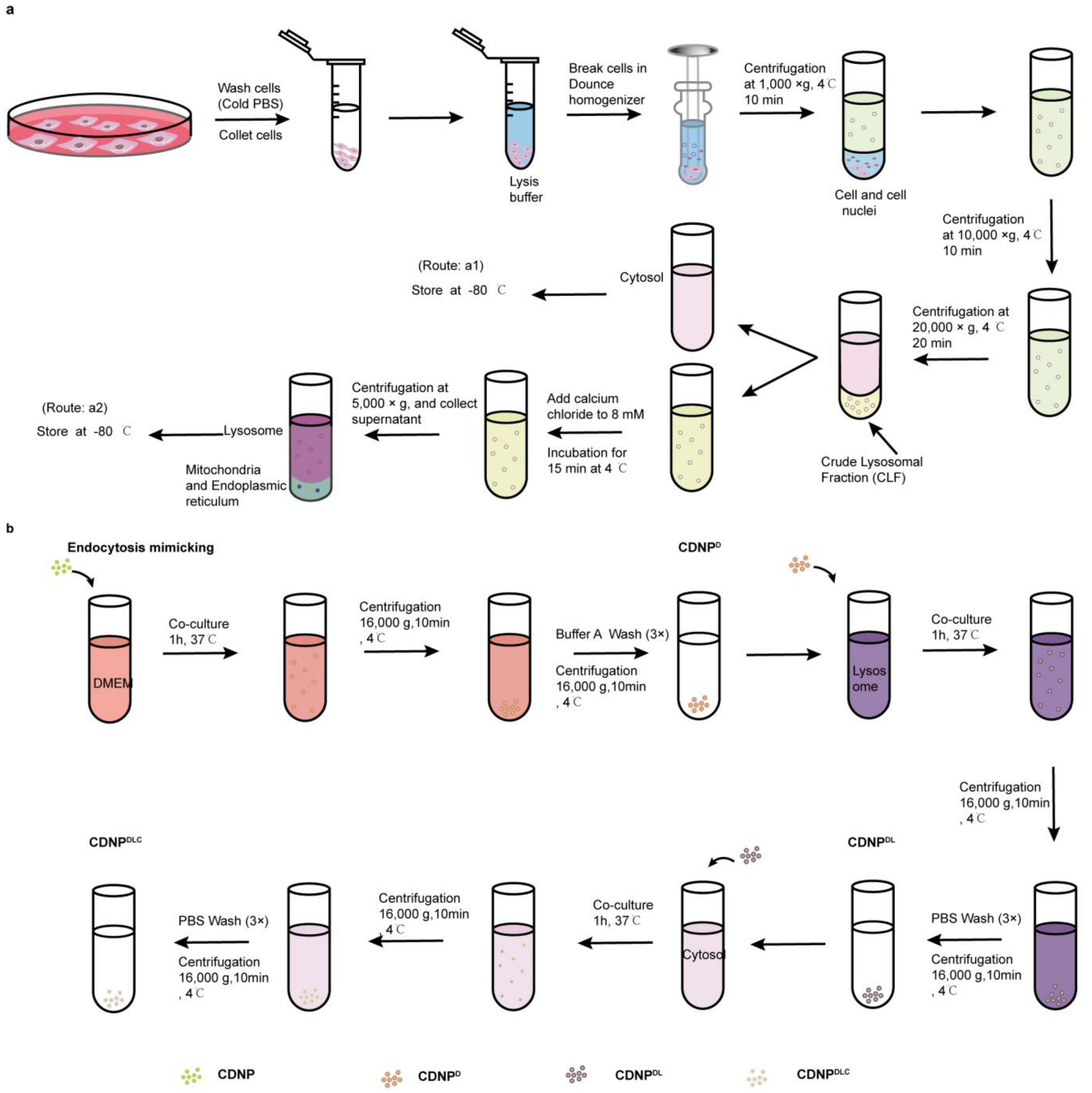

Supplementary Figure 10. Flowchart of nanoparticle (NP)-corona complex preparation. a Flowchart of (a1) cytosol and (a2) lysosome from the HepG2 cells. **b** Flowchart of NP incubation in the extracted bio-fluids to mimic the endocytosis of the CDNP.

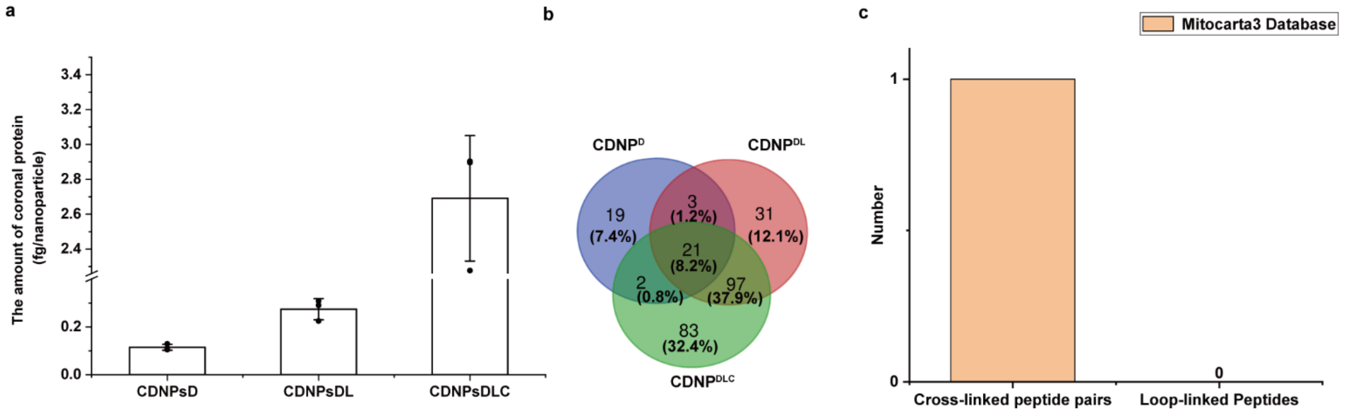

Supplementary Figure 11. Assessment of nanoparticle (NP)-corona complexes. a Quantification (protein femtogram [fg] per particle) of the protein amount within the three specific protein coronas (black). Data in (a) was represented as mean values ± SD, n = 3 technical triplicates. **b** The proteins were identified using LC-MS/MS in the respective NP coronas under the three different incubation conditions, based on the biological processes of the CDNP before reaching the mitochondria of the HepG2 cells. Venn diagram of the numbers of identified proteins on the CDNP^{D} (blue), CDNP^{DL} (red), and CDNP^{DLC} (green) surfaces. Detailed values for all individual proteins were available in the Supplementary Table 3. **c** Evaluation of the number of cross-links on the CDNP^{DLC} surfaces (orang). Source data are provided with this paper.

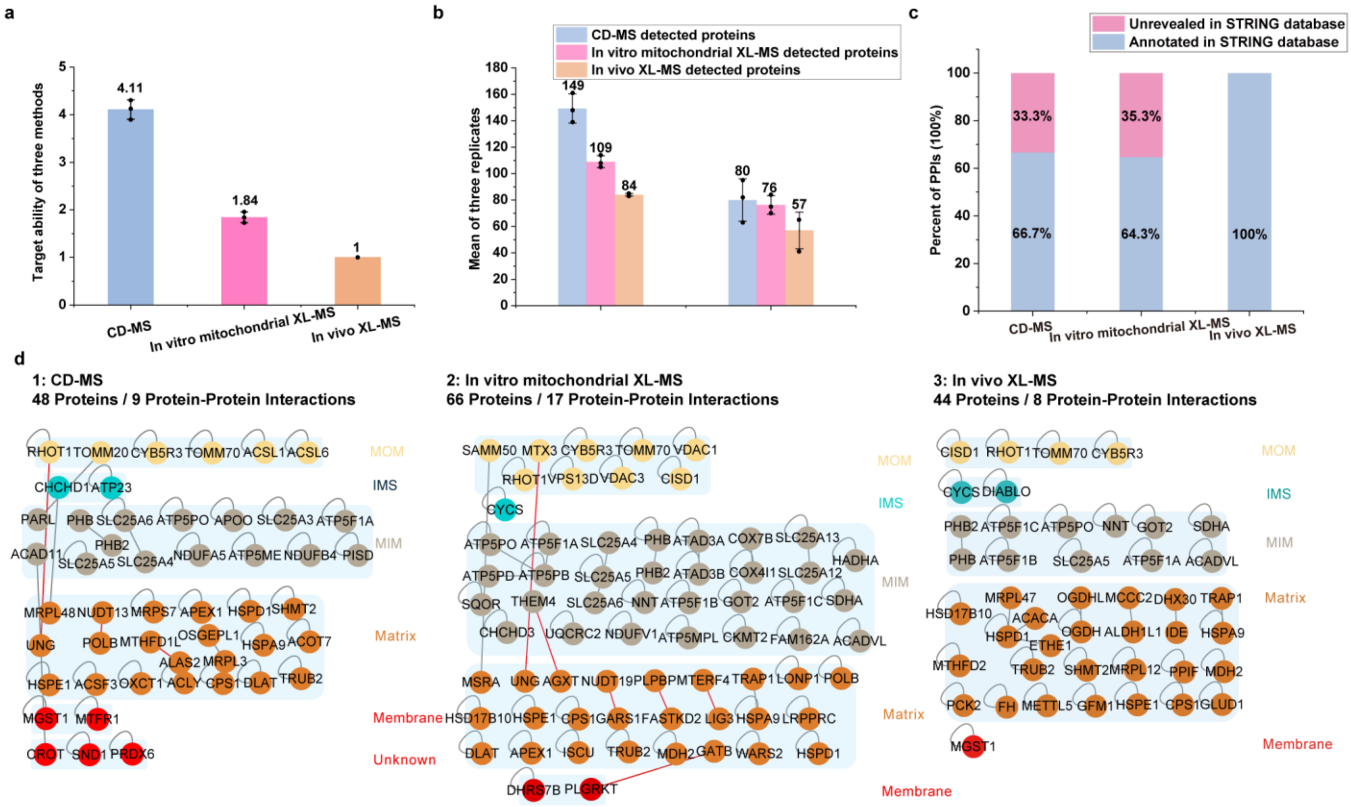

Supplementary Figure 12. Evaluation of the effect of CD-MS labeling mitochondrial proteins. a Target ability of the three methods (Including: CD-MS (sky blue); In vitro mitochondrial XL-MS (light magenta) and In vivo XL-MS (orange)), target ability = mitochondrial fraction percent (α: DSS modification mitochondria peptides/all peptides)/in vivo cross-linking coupled to mass spectrometry (XL-MS) (α). Data in (a) was represented as mean values ± SD, n = 3 technical triplicates. **b** The number of the detected mitochondrial protein in Mitocarta 3.0 and the Homo sapiens database in three replicates for the three methods (Including: CD-MS (sky blue); In vitro mitochondrial XL-MS (light magenta) and In vivo XL-MS (orange)). Data in (b) was represented as mean values ± SD, n = 3 technical triplicates. **c** The inter-protein-protein interactions (PPIs) detected using the three methods matched with the protein interactions reported in the STRING database, and the proportion was calculated. The percentage of unreported interactions was shown in light magenta, and the percentage of reported interactions was shown in gray. **d** According to SubMitoLocalization of the protein in

MitoCarta3, all cross-linked peptides detected in the three replicates in three methods were classified and displayed. MitoCarta3_SubMitoLocalization: mitochondrial outer membrane (MOM), intermembrane space (IMS), mitochondrial inner membrane (MIM), matrix, mitochondrial membrane (membrane), and unknown sub-mitochondrial localization (unknown). Nodes represented the individual proteins, while the lines represented all cross-links identified between two proteins. The lines linking unreported interactions were shown in red, and the lines linking the reported interactions were shown in grey. The proteins of MOM were colored in yellow; the proteins of IMS were colored in dark blue; the proteins of MIM were colored in grey; the proteins of the matrix were colored in orange; and the membrane and unknown proteins were colored in red. Source data are provided with this paper.

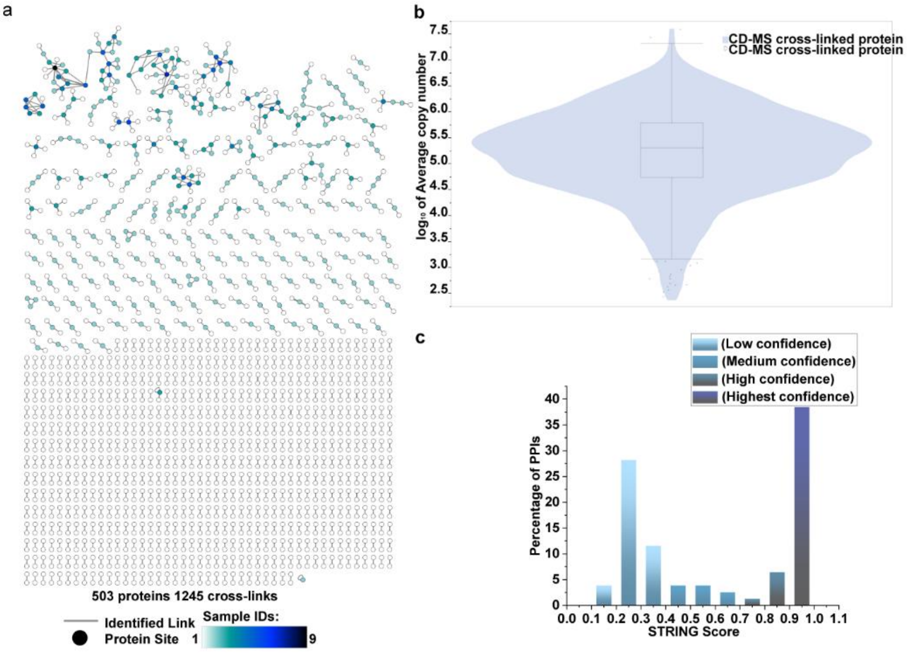

Supplementary Figure 13. Analysis of the CD-MS-labeled mitochondrial cross-linked peptides. a Analysis of the cross-linked peptide results identified in all fractionations in a global view. Protein interactions were determined through the targeted delivery of cross-linkers to the mitochondria in the HepG2 cells via NPs for cross-linking. The nodes represented individual protein sites, and the lines represented all cross-links identified between two proteins. Each node was colored based on the number of samples in which each protein interaction was observed. An interactive network depicting site-to-site interactions is available in Source Data file. **b** Violin plot of the distribution of the copy number of proteins detected in all cross-linked peptides spanned a dynamic range of five orders of magnitude, which correlated with the protein expression abundance. For boxplots, the center line, boxes and whiskers represent the median, inner quartiles, and rest of the data distribution (Minima (2.437), maxima (7.591), center (5.311), bounds of box (Q1: 4.740; Q3: 5.788)), respectively. Data in (b) was represented as mean values ± SD, n = 3 biological triplicates. **c** Inter-link identified protein interactions and scored in the string database. Low confidence = 0.15–0.4 (light blue); medium confidence = 0.4–0.7 (blue)); high confidence = 0.7–0.9 (dark blue); highest confidence = 0.9–1 (purple black). Source data are provided with this paper.

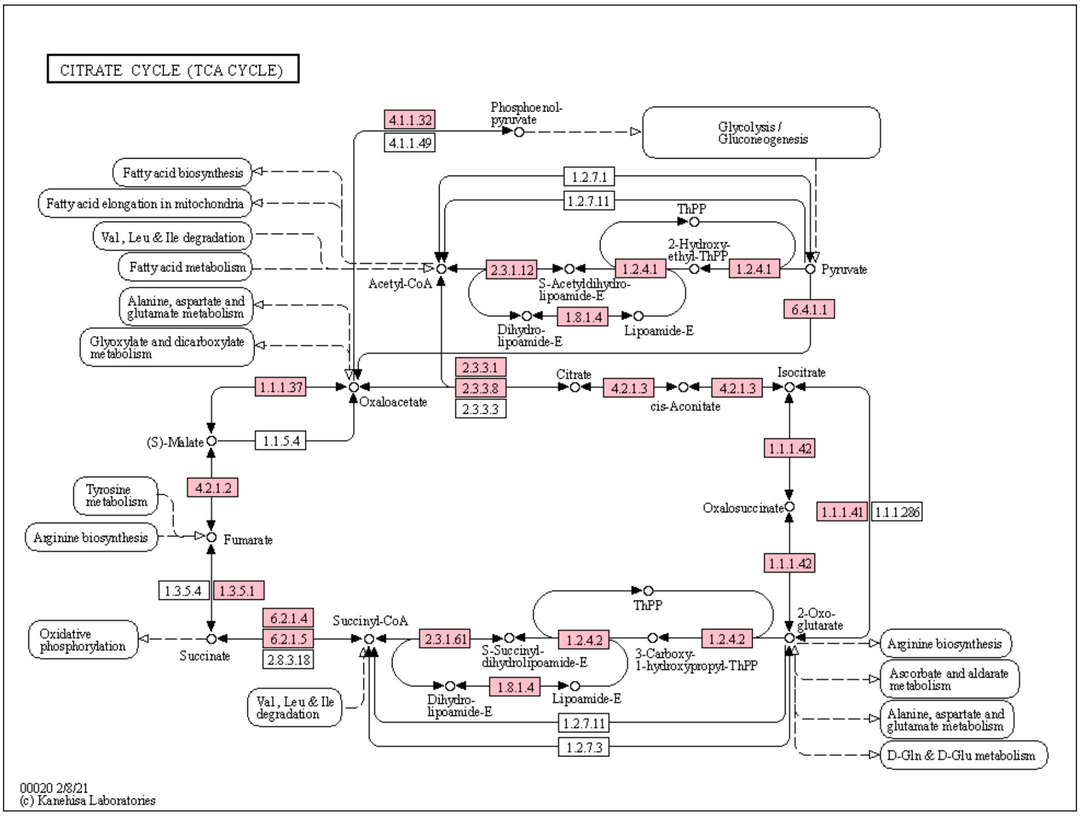

Supplementary Figure 14. Pathway analysis of the identified tricarboxylic acid cycle (TCA cycle) proteins using the KEGG mapper. The KEGG mapper was used to analyze the biological pathways of the proteins in the TCA cycles identified by cross-linked peptides. The protein subunits identified by the cross-linked peptides were shown in pink. The proteins from bacteria or archaea were shown in white (abbreviation: B/A).

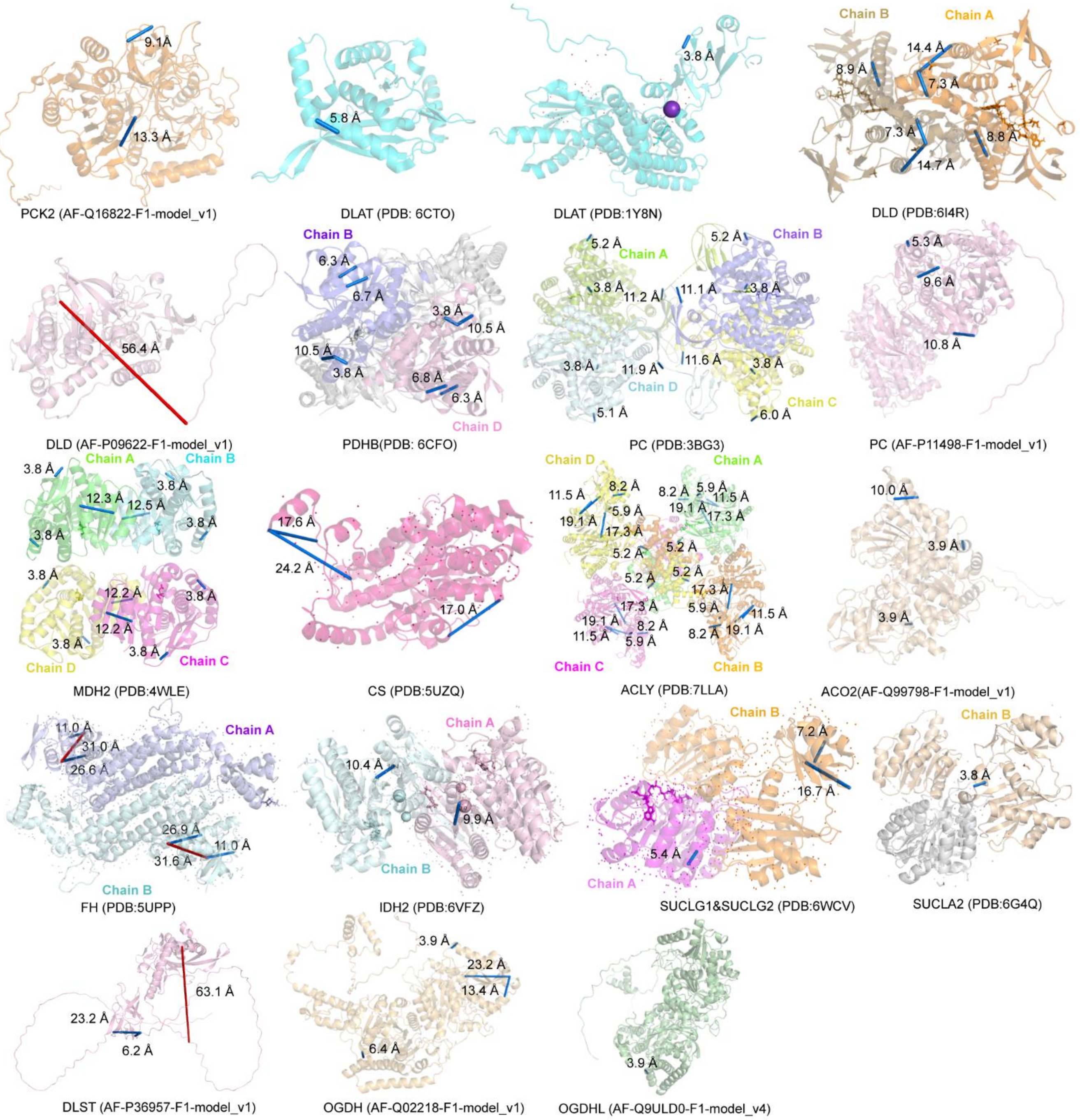

Supplementary Figure 15. Structural match of the detected cross-linked peptides of tricarboxylic acid cycle (TCA cycle) proteins. Each protein chain was colored in a different color. No structural information was available for ACO2 (wheat), PCK2 (orange), OGDHL (pale-green) and the unresolved region of the reported protein structure, including PC (Lys-104 to Lys-107) (light pink), DLD (Lys-104 to Lys-267, Lys-1 to Lys-273, Lys-101 to Lys-104, and Lys-127 to Lys-132) (light pink), DLST (Lys-145 to Lys-154, Lys-217 to Lys-272, and Lys-152 to Lys-154) (light pink), and OGDH (Lys-899 to Lys-981, Lys-532 to Lys-534, Lys-999 to Lys-1000, and Lys-899 to Lys-907) (light orange). Therefore, the detected cross-linked peptides from the above proteins were mapped to AlphaFold model. Ala-2 to Lys-401 in SUCLG2 could not be identified because no experimental or predicted structural models were available. The protein sites involved in the remaining cross-linked peptides matched the structures reported in PDB database. DLAT (PDB: 6CTO & 1Y8N) (cyan), DLD (PDB: 6I4R) (Chain a: orange and Chain b: brown), PDHB (PDB: 6CFO) (Chain d: light pink and Chain b: purple), PC (PDB: 3BG3) (Chain a: limon, Chain b: purple, Chain c: yellow and Chain d: pale-cyan), MDH2 (PDB: 4WLE) (Chain a: green, Chain b: pale-cyan, Chain c: light magenta and Chain d: yellow), CS (PDB: 5UZQ) (light magenta), ACLY (PDB: 7LLA) (Chain a: green, Chain b: orange, Chain c: magenta and Chain d: yellow), FH (PDB: 5UPP) (Chain a: purple and Chain b: pale-cyan), IDH2 (PDB: 6VFZ) (Chain a: light pink and Chain b: pale-cyan), SUCLG1 & SUCLG2 (PDB: 6WCV) (Chain a: magenta and Chain b: orange), and SUCLA2 (PDB: 6G4Q) (Chain b: wheat). Cross-links mapped on the structure within

30 Å were shown in blue, and cross-links mapped on the structure exceeded 30 Å were shown in red. Source data are provided with this paper.

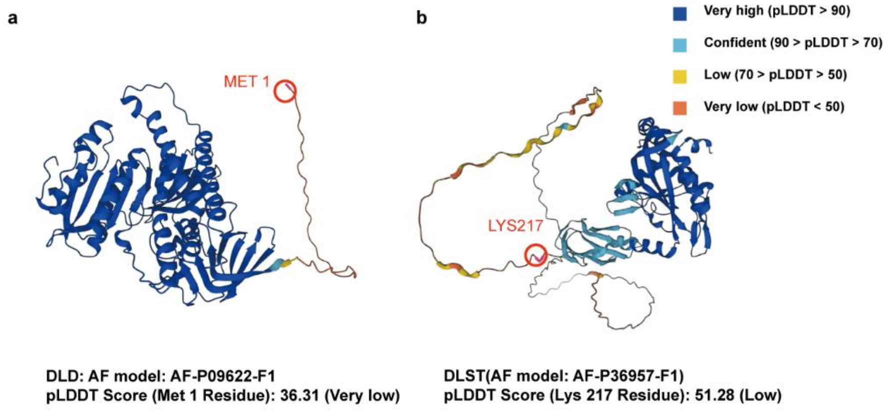

Supplementary Figure 16. AlphaFold prediction model. a AlphaFold prediction model for DLD. The prediction was colored by the model confidence band; **b** AlphaFold prediction model for DLST, where the prediction was colored by the model confidence band.

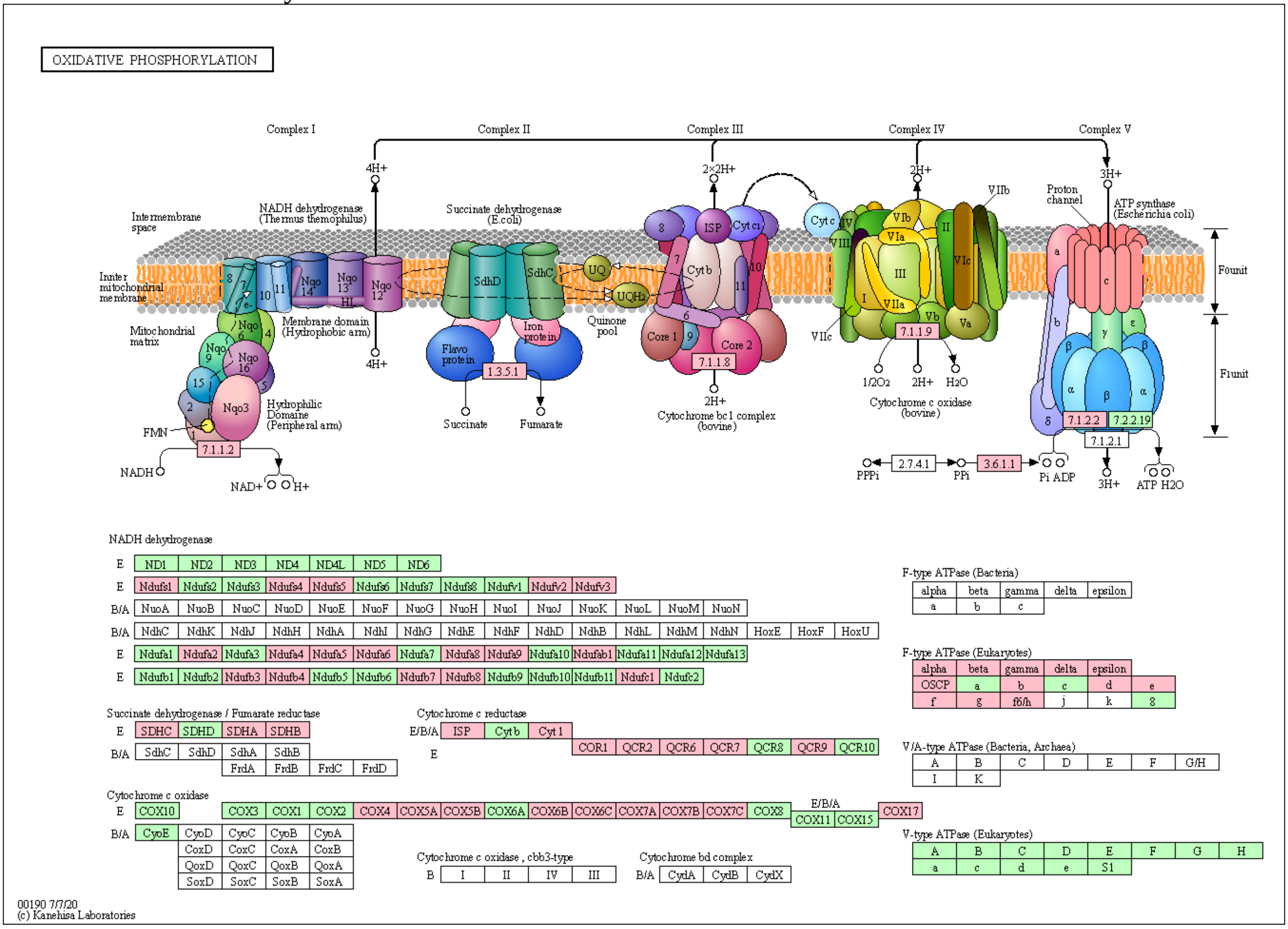

Supplementary Figure 17. Pathway analysis of the identified oxidative phosphorylation (OXPHOS) proteins using KEGG mapper. KEGG mapper was used to analyze the biological pathways of the proteins in OXPHOS, which were identified by cross-linked peptides. The protein subunits identified by the cross-linked peptides were shown in pink, and those not identified were shown in green. The proteins from bacteria or archaea were shown in white (abbreviation: B/A).

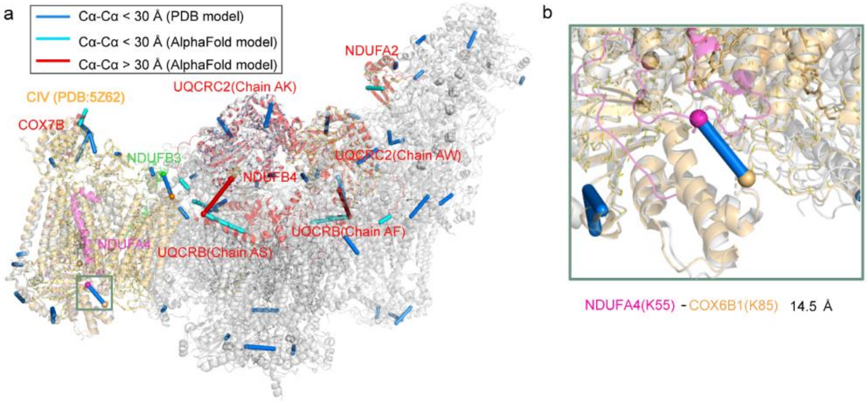

Supplementary Figure 18. Analysis of the cross-linked peptides of the oxidative phosphorylation (OXPHOS) proteins identified in all fractionations. a The detected cross-linked sites were mapped on the supercomplex ($SCI_1III_2IV_1$) structure, and the cross-links were positioned on the Cα-Cα link of the subunit residues in the complex structure (PDB code: 5Z62 & 5XTH). The subunits of 5XTH) were shown in grey, and the subunits of 5Z62 were shown in orange. The AlphaFold model aligned with the $SCI_1III_2IV_1$ was shown in red (details are available in Methods section) (including COX7B, NDUFB4, NDUFA2, UQCRC2, and UQCRB), where the cross-links were positioned on the Cα-Cα link of the subunit residues of the AlphaFold model. Cross-linked peptides mapped on the structure within 30 Å were shown in blue (PDB model) or cyan (AlphaFold model), and cross-linked peptides mapped on the structure exceeded 30 Å were shown in red. **b** NDUFA4 of $SCI_1III_2IV_1$ was colored in magenta, and the cross-linked Cα-Cα distance between the lys-55 of NDUFA4 and the lys-85 of COX6B1 was measured, as shown in red. Source data are provided with this paper.

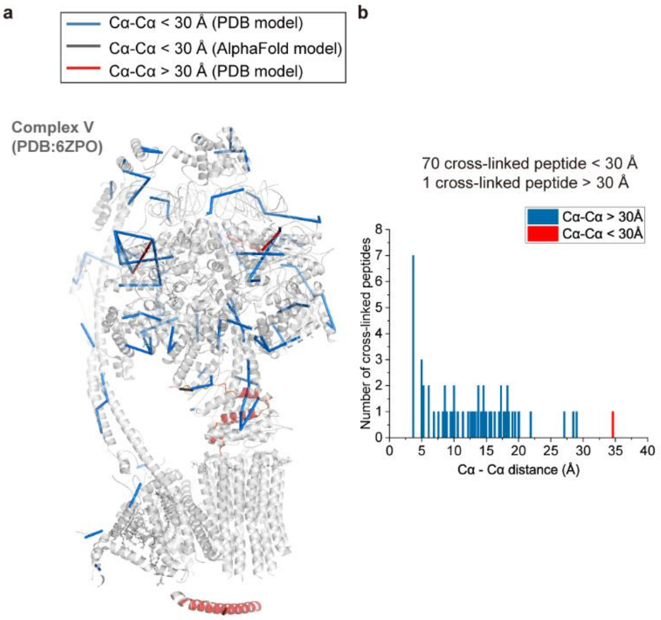

Supplementary Figure 19. Analysis of the cross-linked peptides of ATP synthase (complex V) identified in all fractionations. a The detected cross-linked sites were mapped on the electron microscopy structure of complex V (PDB code: 6ZPO) from Bos taurus in monomer state 1. The structure of complex V was colored in grey. The Cα-Cα distance between the two residues was shown as a line, with blue (PDB model) or grey (AlphaFold model) lines representing cross-links with a Cα-Cα distance of less than 30 Å. Furthermore, the red line represents links with a Cα-Cα distance of more than 30 Å. Human ATP5F1B K133 corresponded to R83 in the bovine structure, and human ATP5F1C K189 corresponded to R164 in the bovine structure link the proteins. The AF-P56381-F1-model_v4 was aligned to ATP5F1E (subunit i) of structure complex V in red (RMSD = 0.734, 42 to 42 atoms). The AF-P56385-F1-model_v1 was aligned to ATP5ME (subunit e) of structure complex V in red (RMSD = 0.629, 39 to 39 atoms). **b** The histograms showed the length distribution of all distance restraints on the complex V structures in Supplementary Fig. 19a after docking. Cross-linked peptides mapped on the structure within 30 Å were shown in blue, and cross-linked peptides mapped on the structure exceeded 30 Å were shown in red. Source data are provided with this paper.

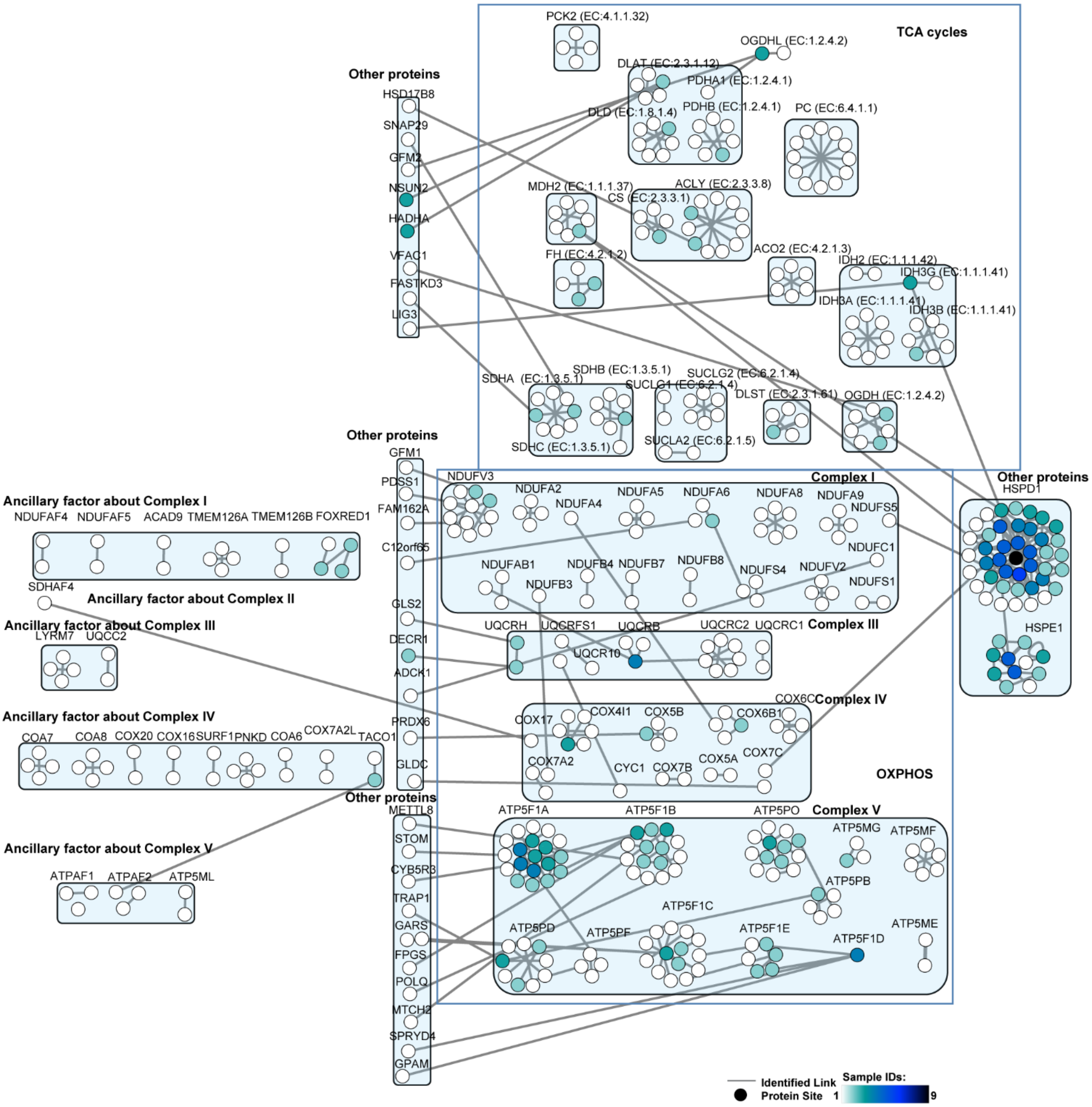

Supplementary Figure 20. Analysis of the cross-linked peptides of the tricarboxylic acid cycle (TCA cycle) and oxidative phosphorylation (OXPHOS) identified in all fractionations. The cross-linked peptides involved in the TCA cycle or OXPHOS were selected from Supplementary Fig. 13a and shown separately, specifically the TCA enzymes, OXPHOS enzymes (complex I–V), other proteins that interacted with the TCA enzymes or OXPHOS enzymes, and the ancillary of proteins involved in the assembly of complex proteins in the OXPHOS enzymes. Each node was colored based on the number of samples in which each protein interaction was observed, and an interactive network depicting site-to-site interactions is available in Source Data file. Source data are provided with this paper.

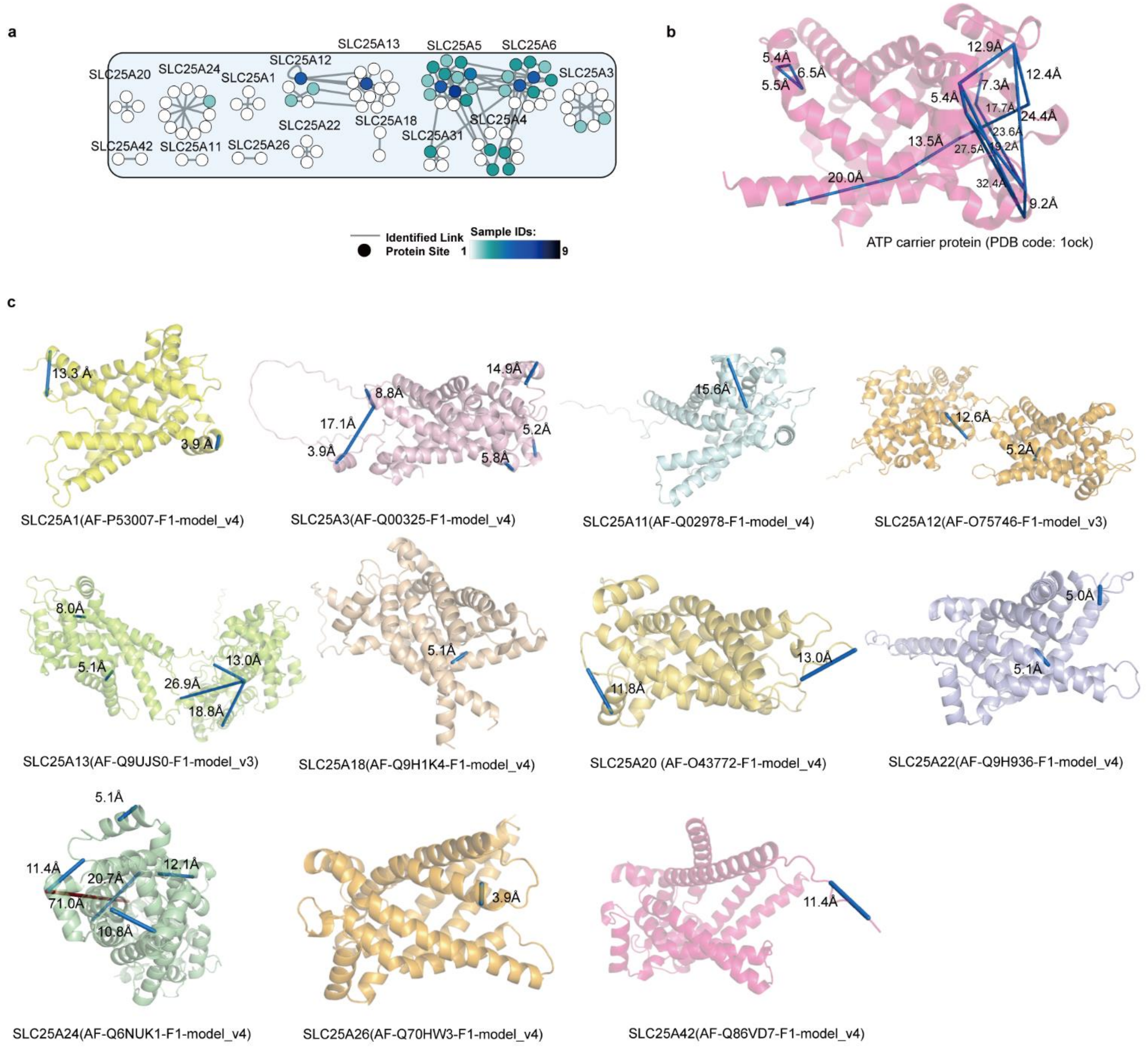

Supplementary Figure 21. Structural match of the detected cross-linked peptides of the SLC25 family. **a** The cross-linked peptides involved in the TCA cycle proteins were selected from Supplementary Fig. 13a and displayed individually. Each node was colored based on the number of samples in which each protein interaction was observed, and an interactive network depicting site-to-site interactions is available in Source Data file. **b** The ATP carrier protein heart isoform T1 (PDB code: 1ock), with the structure of the ATP carrier protein heart isoform T1 colored in magenta. The human cross-linked sites were mapped on the crystal structure of malonamidase E2 from *Bradyrhizobium japonicum*. The line representing the Cα-Cα connection of the cross-linked residues was shown in blue. **c** Each protein chain was colored in a different color. No structural information was available for SLC25A1 (yellow), SLC25A3 (light pink), SLC25A11 (pale-cyan), SLC25A12 (orange), SLC25A13 (limon), SLC25A18 (wheat), SLC25A20 (yellow orange), SLC25A22 (light blue), SLC25A24 (pale-green), SLC25A26 (orange), and SLC25A42 (hot pink). Thus, the detected cross-linked peptides from the above proteins were mapped to the AlphaFold predicted model. Some of the cross-linked peptide residues detected were located in the unresolved region of the reported protein structure, including SLC25A25 (Lys-74 to Lys-243, Lys-190 to Lys-477, Lys-320 to Lys-243, Lys-228 to Lys-437, Lys-333 to Lys-336, and Lys-268 to Lys-276), SLC25A12 (Lys-234 to Lys-244 and Lys-403 to Lys-406), and SLC25A13 (Lys-235 to Lys-5, Lys-235 to Lys-245, Lys-235 to Lys-312, Lys-405 to Lys-408, and Lys-353 to Lys-580). Thus, the detected cross-linked peptides from the above proteins were mapped to the AlphaFold model. Cross-links mapped on the structure within 30 Å were shown in blue, and cross-links mapped on the structure exceeded 30 Å were shown in red. Source data are provided with this paper.

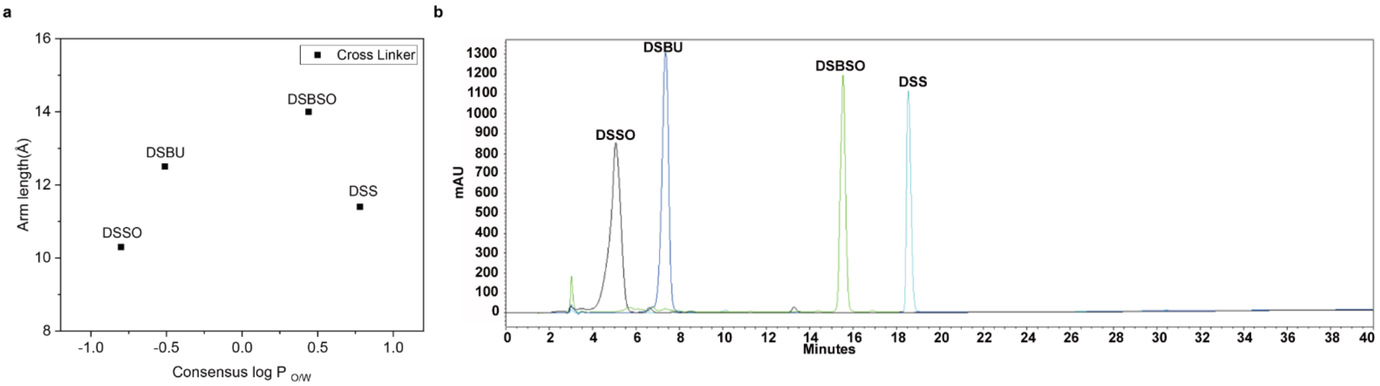

Supplementary Figure 22. Hydrophobic/hydrophilic properties of cross-linkers. a The n-octanol-water partition coefficient (log Po/w) of the cross-linker (Including: disuccinimidyl sulfoxide (DSSO), disuccinimidylsuberate (DSS), DSBU (disuccinimidyl dibutyric urea; formerly BuUrBu)). **b** The chromatogram of the cross-linker analyzed on C18. Source data for a are provided in Source Data file.

Supplementary Table 1. Size, PDI, and zeta potential variations of the three nanoparticles (NPs) in Fig. 2b.

	Diameter (nm)	PDI	Zeta potential (mv)
10DSS-DDAB@PLGA/Kolliphor EL NPs-1	321.8 ± 5.9	0.080	37.5 ± 0.4
10DSS-DDAB@PLGA/Kolliphor EL NPs-2	307.7 ± 4.6	0.108	34.8 ± 0.5
10DSS-DDAB@PLGA/Kolliphor EL NPs-3	316.7 ± 2.9	0.100	33.2 ± 0.6
40DSS-DDAB@PLGA/Kolliphor EL NPs-1	297.2 ± 2.4	0.238	40.0 ± 1.0
40DSS-DDAB@PLGA/Kolliphor EL NPs-2	274.3 ± 2.4	0.112	42.1 ± 0.1
40DSS-DDAB@PLGA/Kolliphor EL NPs-3	287.4 ± 1.4	0.043	42.6 ± 0.5
70DSS-DDAB@PLGA/Kolliphor EL NPs-1	275.7 ± 5.5	0.067	38.5 ± 2.5
70DSS-DDAB@PLGA/Kolliphor EL NPs-2	270.7 ± 4.1	0.104	37.7 ± 0.3
70DSS-DDAB@PLGA/Kolliphor EL NPs-3	281.7 ± 0.5	0.126	37.5 ± 1.9

Supplementary Table 2 . In vitro disuccinimidylsuberate (DSS) release experiment of 70DSS-dimethyldioctadecylammonium bromide (DDAB) @ poly (lactic-co-glycolic acid) (PLGA) / nanoparticles (NPs). The amount of DSS released by 70DSS-DDAB@PLGA/Kolliphor EL NPs in PBS (0.01 M, pH = 7.2 (1% DMSO (v/v)) at different times was measured by high-performance liquid chromatography (HPLC).

Time (h)	0.5	1	1.5	2	2.5	3	3.5
Cumulative release (%)	4.32	6.50	8.78	11.3	13.6	15.5	17.7
Time (h)	4	4.5	5	5.5	6	7.5	8
Cumulative release (%)	20.4	22.1	24.2	26.8	28.7	32.6	34.5

Supplementary Table 3. The adsorbed proteins on the CDNP^{D}, CDNP^{DL}, and CDNP^{DLC} surfaces, corresponding to the numbers in Supplementary Fig. 11b. The proteins from Homo sapiens are shown in black (from HepG2 cells); bovine proteins are shown in red (from the DMEM medium with 10% fetal bovine serum).

	21	97			83				31				3	2	19	
CDNPs-DLC	CD44	HSPD1	APEX1	SQSTM1	RPL10	RPLP0	HNRNPA1	ISG15						C4		
	H2BC4	ACTB	PDIA6	SNRPD3	RPS25	PCBP1	RPS20	RPL39P5						TUBA1B		
	ITIH3	YBX1	TFRC	HSPA8	RPS17	VAT1	RPL35	TMED10								
	HSPA5	ANXA2	RPL6	PDIA3	ERGIC1	RPS4X	RPS10	RRM1								
	APOA1	SUMF2	SPTBN1	RPL11	RPS3A	RPL36	PHGDH	S100A10								
	GAPDH	ENO1	MPDU1	TUBB4B	RPN2	RPS3	RPL27A	RAB7A								
	Q9TRI1	RPL12	RPL13A	RPL9	RPL23	RPL3	RPS19	H3C1								
	TUBA1C	HYOU1	SERPINH	SPCS2	CRIP1	SAR1A	M6PR	RPL31								
	PKM	TUBB	RACK1	RPSA	CNPY2	ITGB1	RPS13									
	VIM	HSPB1	ILF3	RPL8	SNRPG	RPL30	RPS29									
	ACTG1	PPIB	RPS14	FBL	RPL10A	RPL7A	RHOC									
	AMBP	PRKCSH	RPS5	PLP2	RPL17	RPS7	PTBP1									
	KRT1	RRBP1	SRP14	H2AC6	CKB	CALR	CNIH4									
	KRT18	RPS18	RPN1	EEF1G	EIF3F	ACTC1	RAN									
	EEF1A1P5	RPLP1	ATP5F1B	H3C15	VDAC1	NONO	LCT									
	HBB	PPIA	MT-CO2	HNRNPK	G3BP1	RPLP2	TUBB8									
	H1-2	SCAMP3	RPS24	RHOG	RPS12	RPL27	CYB5B									
	VTN	LMAN1	HNRNPU	SSR4	HSPA7	DAD1	PCBP2									
	KRT17	HNRNPH1	PLEC	BANF1	CCT2	RPL38	H3-3B									
	KRT7	TXNDC5	HSP90AB	HNRNPC	RHEB	RPS6	EEF1D									
	APOE	HLA-C	PRDX4	EIF4A1	ARL6IP5	KRT8	BSG									
		FAU	VCP	ALPP	RPL35A	RPL32	MYL12A									
		MYH9	SLC3A2	P4HB	XRCC6	RPL22	RAB6A									
		RPL19	RPS16	CKAP4	FUS	RPS9	RPS15A									
		EEF2	ALDOA	TMX1	VASP	AHSA1	RPL4									
CDNPs-DL		HNRNPM	RPL18	RPL13					GAPDHS	PSMD11	AK2	MFF	PGAM2			
		RPL28	CANX	COLGALT1					NDUFS6	NEFM	ERP29	ERP44	KRT5			
		SEC61G	SRSF3	PARP1					PDHA1	SEC22B	SFPQ	HAX1	PRSS1			
		HSD17B4	HSP90B1	RPL15					NDUFS2	NDUFS1	TOMM6	PHB1				
		RPL7	NCL	ATP5F1A					RNPS1	UQCRFS1	LAMTOR3	PF4				
		NPM1	RPL14	GNAO1					TMEM43	SYNCRIP	SUCLA2	TOP1				
		DDX39A	H4C1						PHB2	DDX18	H3-7	EDF1				
		GANAB	HNRNPA2B1						MDH2	LETM1	ARL8B					
CDNPs-D															CREG1	OXNAD1
															FMOD	KRT9
															PARVG	S100A8
															OR1L6	MRPS21
															XP32	CCNC
															F13B	F2
															KRT14	KRT10
															LUM	NEFH
															KPRP	S100A9
															CLU	

Supplementary Table 4. A comparison of different methods for studying mitochondrial information. Cross-linking information on mitochondrion from different studies in terms of the number of detected inter-peptides, number of intra/loop peptides, percentage of inter-peptides, number of proteins, unique PPI, FDR, etc.

**Author**	**Inter peptide number**	**Intra/Loop peptide number**	**Total cross-links**	**Inter-peptide percent (%)**	**Protein number**	**Unique PPIs**	**FDR (%)**	**Database**	**Cross-linker**	**The length of the maximum Cα-Cα distance (Å)**	**MS**	**Fractionation**	**Search engine**	**Species**	**Reference**
**Schweppe et al.**	**701**	**1851**	**2427**	**29**	**327**	**236**	**5**	**Mitocarat2.0**	**PIR (BDN-NHP)**	**43**	**Velos-FT**	**SCX**	**ReACT**	**Mouse**	1
**Liu① et al.**	**2040**	**1282**	**3322**	**61**	**359**	**608**	**2**	**Mitocarat2.0**	**DSSO**	**30–40**	**Lumos**	**SCX**	**XlinkX v2.0**	**Mouse**	2
**Liu② et al.**	**1419**	**1454**	**2873**	**49**	**290**	**357**	**2**	**Mitocarat2.0**	**DSSO**	**30–40**	**Lumos**	**SCX**	**XlinkX v2.0**	**Mouse**	2
**Linden③ et al.**	**359**	**1741**	**2100 ⑤**	**17**	**263**	**139**	**1**	**A database with the 400 most abundant proteins (top400)**	**BS3**	**30**	**Lumos**	**SEC**	**pLink1**	***S. cerevisiae***	3
**Linden④ et al.**	**265**	**1522**	**1787 ⑤**	**15**	**260**	**113**	**1**	**A database with the 400 most abundant proteins (top400)**	**BS3**	**30**	**Lumos**	**SEC**	**pLink1**	***S. cerevisiae***	3

**Ryl et al.**	**152**	**5367**	**5518 ⑥**	**2.8**	**792**	**152**	**5**	**MitoCarta 2.0 database**	**DSS**	**30**	**Lumos**	**SCX −SD −SEC**	**Xi (version 1.6.731)**	**Human**	4
**Chen et al.**	**158**	**394/651**	**1203**	**13**	**501**	**152**	**1**	**Mitocarta3.0 database**	**DSS**	**30**	**QE**	**RPLC**	**pLink2**	**Human**	
	**①. Native mitochondrion isolated from mouse heart tissue.**<br>**②. Mitochondrion isolated from mouse heart tissue and treated with salt.**<br>**③. Mitochondrion isolated from *S. cerevisiae* grown on glycerol.**<br>**④. Mitochondrion isolated from *S. cerevisiae* grown on glucose.**<br>**⑤. Datasets were filtered by removing ambiguous identifications, with cross-links only supported by a single cross-linked peptide; spectrum match (CSM) and/or with a log10-transformed pLink 1 spectrum score below 4.**<br>**⑥. Unique cross-linked residue pairs.**														

Supplementary Reference

1. Schweppe D.K, *et al.* Mitochondrial protein interactome elucidated by chemical cross-linking mass spectrometry. *Proc Natl Acad Sci U S A* **114**, 1732-1737 (2017).
2. Liu F, Lossl P, Rabbitts B.M, Balaban R.S, Heck A.J.R. The interactome of intact mitochondria by cross-linking mass spectrometry provides evidence for coexisting respiratory supercomplexes. *Mol Cell Proteomics* **17**, 216-232 (2018).
3. Linden A, *et al.* A Cross-linking Mass Spectrometry Approach Defines Protein Interactions in Yeast Mitochondria. *Mol Cell Proteomics* **19**, 1161-1178 (2020).
4. Ryl P.S.J, *et al.* In Situ Structural Restraints from Cross-Linking Mass Spectrometry in Human Mitochondria. *J Proteome Res* **19**, 327-336 (2020).
